# SPARCL1 Influences Bovine Skeletal Muscle-Derived Satellite Cell Migration and Differentiation through an ITGB1-Mediated Signaling Pathway

**DOI:** 10.3390/ani10081361

**Published:** 2020-08-06

**Authors:** Yuxin Wang, Shuaiyu Liu, Yunqin Yan, Shufeng Li, Huili Tong

**Affiliations:** Laboratory of Cell and Developmental Biology, Northeast Agricultural University, Harbin 150030, China; wangyuxin202071@163.com (Y.W.); liushuaiyu2020@163.com (S.L.); yanyunqin@sohu.com (Y.Y.); lishufeng19771019@outlook.com (S.L.)

**Keywords:** SPARCL1, migration, differentiation, bovine, skeletal muscle-derived satellite cells, ITGB1

## Abstract

**Simple Summary:**

It is known that cell migration and differentiation have a very important yet simple basis for muscle development and muscle disease treatment. Secreted protein acidic and rich in cysteine like 1 (SPARCL1), one of the components of extracellular matrix, has been proved to regulate bovine skeletal muscle-derived satellite cell differentiation. However, the exact mechanism is not yet clear. This study reveals that SPARCL1 promotes muscle-derived satellite cell early differentiation through integrin β1, thereby providing a new insight into the role of SPARCL1 in muscle development.

**Abstract:**

As an extracellular matrix protein, secreted protein acidic and rich in cysteine (SPARC)-like 1 (SPARCL1) is involved in various cell functions. It was previously implicated in bovine skeletal muscle-derived satellite cell (MDSC) differentiation; however, the underlying mechanism remains unknown. In this study, immunoprecipitation and mass spectrometry revealed that integrin β1 (ITGB1) combines with SPARCL1. Further, co-immunoprecipitation demonstrated that SPARCL1 interacts with ITGB1. Cell scratch assays explored the influence of SPARCL1 on MDSC migration through ITGB1. In addition, desmin staining for myotube fusion rate and MyoD protein expression results showed that SPARCL1 promotes MDSC early differentiation through ITGB1. Furthermore, Western blotting results demonstrated that SPARCL1 regulates the expression of p-FAK, p-paxillin, vinculin, Cdc42, and Arp2/3 through ITGB1. These findings indicate that SPARCL1 may influence bovine MDSC migration and differentiation through an ITGB1-mediated cell signaling pathway. Herein, we elucidated the mechanism through which SPARCL1 affects MDSC differentiation. Our results provide insight into the molecular mechanism of muscle development and may in the future facilitate skeletal muscle regeneration and treatment.

## 1. Introduction

SPARC (osteonectin or BM-40), as a member of the secreted protein acidic and rich in cysteine (SPARC) family of acidic secretory glycoprotein, is known in a variety of cell functions, including cell proliferation, cell turnover, cell differentiation and tissue repair [1,2,3]. SPARC has been reported to play important roles in skeletal muscle myoblast differentiation [4,5]. SPARCL1 (SPARC-like protein 1, also known as ECM2, Hevin, MAST 9, or SC1), also belongs to SPARC family of matricellular proteins, it shares a follistatin-like module and an extracellular Ca^2+^ binding domain in that of SPARC in mouse [6]. A report showed that SPARCL1 and SPARCL1 may share the similar biological function such as the antagonist of cell adhesion [3]. Furthermore, in previous studies, we also found SPARCL1 played important roles in C2C12 cell differentiation and bovine skeletal muscle-derived satellite cells (MDSCs) differentiation [7,8]. However, the underlying mechanism of SPARCL1 regulating bovine MDSC differentiation remains unclear.

It is known that satellite cells (SCs) localized between sarcolemma and basal lamina in skeletal muscle. SCs as a type of stem cell associated with muscle development, growth, and regeneration [9]. The quiescent state of SCs is broken up when muscle injury or other signal stimuli, then SCs are activated to differentiate. SCs undergo a series of processes such as proliferation, cell cycle withdrawal, migration, and fusion to reconstruct the myotubes and then muscle fibers [10]. Cell migration is a critical process for SCs differentiation, myotubes formation has been shown to be reduced by the inhibition of cell migration [11]. In addition, the development of skeletal muscle is a highly sophisticated and complex biological processes and it regulated by multiple factors including extra-cellular matrix (ECM) [12]. Whereas, integrin-mediated cell adhesion to the ECM is implicated in the control of proliferation, survival, migration and differentiation of myoblasts [13,14].

Integrin β1 (ITGB1) is a member of the β sub-family, which forms dimers with different α subunits. A recent study by our team showed that the inhibition of ITGB1 and focal adhesion kinase (FAK) repressed bovine MDSC migration [15]. FAK mediates signals from integrins and plays an essential role in myotube formation, and Integrin/FAK pathway is required for myoblast differentiation by regulating the expression of MyoD and Cdc42 [14]. In cell migration, flaky pseudopods are induced by the Arp2/3 complex [16] after Arp2/3 complex activation by Cdc42 [17]. Branched microfilaments in the pleated pseudopods provide the driving force through polymerization; the cell membrane of the leading edge stretches forward, and the membrane is fixed in a new position by establishing a focal contact between integrin and the ECM [18]. After the leading edge of the cell membrane is spread, only the trailing edge retracts to move the cell forward. The force of the trailing edge retraction is provided by stress fibers [19], and the two ends of the stress fibers are tightly bound to the integrin-mediated adhesion structure-adhesive layer [20]. The formation of the adhesion structure requires the assistance of several adhesion molecules, including p-paxillin, paxillin, vinculin, FAK, and p-FAK, which bind to and recruit the intracellular domain of integrin, establishing adhesion sites with the ECM and allowing cells to migrate [21].

In previous study, SPARCL1 as an extra-cellular matrix protein, influenced on bovine MDSC differentiation [8]. However, its underlying mechanism has not been elucidated. In this study, Co-Immunocoprecipitation (Co-IP) results showed that SPARCL1 may bind with ITGB1. It implies that SPARCL1 may through ITGB1/FAK pathway to regulate bovine MDSC migration and differentiation. Furthermore, whether the adhesion molecules (p-paxillin, paxillin, vinculin, FAK, and p-FAK) and migration related molecules (Cdc42 and Arp2/3 complex) influenced by ITGB1/FAK pathway is worthy to investigate. This study may provide new insights of SPARCL1 regulate skeletal muscle differentiation and development.

## 2. Materials and Methods

### 2.1. Cell Culture and Differentiation

MDSCs were separated from the calf muscles of newborn calves as follows: The musculature of the hind leg of the newborn calf was divided into several small pieces and as many cells as possible were cut out under the basement membrane; the muscles were washed while cutting, cut into small pieces, and the cut meat was put into a 10 mL beaker as soon as possible. A finely minced meat emulsion was prepared and then poured into an Erlenmeyer flask containing type I collagenase (20 mg/L). The digestion was last for 2 h in 37 °C water bath. The digestive product was washed by PBS and repeated centrifugation for 3 times, then it was poured into 0.25% trypsin for a 30 min digestion. Then the digested meat emulsion was poured into a 400-mesh copper sieve, rinsed with PBS, and filtered to obtain the culture mixture including the skeletal muscle satellite cells. The culture mixture was cultured for 2 h and it was transferred into a new polylysine-coated cell flask, the adherent cells remained in original old flask was named as PP1. The culture mixture was continuous cultured for 24 h, then the culture mixture was transferred into another new polylysine-coated cell flask and the adherent cells remained was named as PP2. The same procedure was performed every 24 h and the adherent cells remained was named as PP3 to PP6 separately. PP6 is bovine skeletal muscle satellite cells with higher purity. The cells were cultivated with growth medium to 60–70% confluence, digested with trypsin, and then subcultured.

The study protocol was approved by the Animal Protection Committee of the Northeast Agricultural University, Heilongjiang, China (NEAUEC20200108). Bovine MDSCs were isolated from hindlimb skeletal muscle tissue of newborn Chinese Simmental calves (male) and cultured in high-glucose Dulbecco’s modified Eagle’s medium (Gibco) in 15% fetal bovine serum, 100 U/mL penicillin sulfate, and 100 U/mL streptomycin sulfate at 5% CO_2_ and 37 °C. After cell density reached approximately 90%, the growth culture medium was exchanged with medium supplemented with 2% horse serum to induce MDSC differentiation; when the cell density reached 70%–80%, the cells were used for transfection.

### 2.2. siRNA Interference

RNA interference (RNAi) is a highly efficient and specific gene editing technology that interferes with gene expression. Two *ITGB1* (NCBI Gene ID: 281876) interference fragment pairs were designed by Shenggong Biotechnology Co., Ltd. (Shanghai, China): ITGB1-1, sense 5′-GCGUACAAUUCCCUUUCUUTT-3′ and antisense 5′-AAGAAAGGGAAU-UGUACGCTT-3’, and ITGB1-2, sense 5′-GCUCAGGAAUGUUCACAUUTT-3′ and antisense 5′-AAU-GUGAACAUUCCUGAGCTT-3′. The interference segments were used in combination to silence the expression of *ITGB1*.

### 2.3. Cell Transfection

SPARCL1 activation (VPR-S) and inhibitory vectors (dCas9-S) were constructed by Liu et al. [8] (SPARCL1 is also named as ECM2, thus the effective ECM2 vectors constructed by Liu et al. were used for SPARCL1 activation or inhibition in this study). ZiFiT online software was used to predict SPARCL1 (ECM2) promoter target site (ID: 533916). The fragment “GTCTTTGTTACTATGTGCGG” reported by Liu et al. was synthesized, annealed, and ligated into the BbsI site of the pSPgRNA expression vector. This recombinant plasmid was co-transfected into cells combined with dCas9-VPR or dCas9 plasmid vector to activate or inhibit SPARCL1 expression. Polyethyleneimine (PEI, Sigma, St. Louis, MO, USA) was used for CRISPR transfection. The PEI transfection method was described by Pang et al. [15]. siRNA transfection was performed using LIP2000 according to the manufacturer’s protocol.

### 2.4. Cell Scratch Assay

MDSCs were seeded into six-well plates and cultured to approximately 70% cell density for transfection experiments. Next, a cell-free area was uniformly drawn with a 100 μL pipette tip in each well, cells were washed three times with PBS to remove cell debris, and the medium supplemented with 2% horse serum was changed to induce cell differentiation. Cell migration was observed under a phase contrast microscope (BX43, OLYMPUS, Tokyo, Janpan) at 0 h and 20 h after the scratch. Cell mobility was determined with the following formula: Mobility (%) = (scratch area at T_0_ − scratch area at T)/scratch area at T_0_ × 100.

### 2.5. Western Blotting

MDSCs cultured in six-well plates were washed three times with pre-cooled PBS. The protein was extracted with 100 μL RIPA buffer (Beyotime Biotechnology, Shanghai, China) on ice, and the collected protein was mixed with loading buffer (Beyotime Biotechnology, Shanghai, China) and subjected to high temperature denaturation. Thereafter, electrophoresis was performed with 10% SDS-PAGE gels, and the electrophoresed gels were transferred to PVDF membranes (Millipore Corporation) at a current of 200 mA. The following primary antibodies were used: anti-SPARCL1, anti-ITGB1, anti-p-FAK, anti-FAK, anti-p-paxillin, anti-paxillin, anti-vinculin, anti-Arp2/3, anti-Cdc42, anti-MyoD, and anti-GAPDH (all at dilution 1:500; Bioss Antibodies, China). The membranes were incubated with the primary antibodies overnight at 4 °C, followed by an incubation with a secondary horseradish peroxidase (HRP)-labeled goat anti-rabbit IgG antibody (Bioss Antibodies, China). Protein band intensity was captured and analyzed with the MiniChemi^TM^ 500 Mini Chemiluminescent Imaging and Analysis System (Sage Creation Science, Beijing, China).

### 2.6. Co-Immunoprecipitation

Bovine MDSCs were cultured to a confluence of 70–80% and transferred to a 10 mm cell culture dish. The following day, the differentiation medium was changed and the experiment was conducted 72 h post culturing. Specific steps are listed below:

(1) Cells were washed 3 times with pre-cooled PBS to completely remove the culture medium.
(2) Cells were then collected with 1 mL RIPA buffer (a pre-chilled cell scraper was used to gently scrape the cells from the 10 cm dish, ensuring clean cells). EP tube of the lysate was placed on ice and then on a horizontal shaker for 15–30 min.
(3) Sample was centrifuged at 12,000 rpm for 30 min at 4 °C, and the supernatant was collected, while the precipitate was discarded. A total of 20 μL from the supernatant was taken as the input group.
(4) The supernatant was divided into two parts, 500 μL each, where IgG and protein A/G were added (to remove non-specific binding in the experimental group), along with 1 μg rabbit IgG to the other group and placed on a horizontal shaker at 4 °C for 30 min–2 h.
(5) The experimental group was centrifuged at 2500 rpm for 5 min. The precipitate was discarded while the supernatant was retained.
(6) Approximately 0.2–2 μg primary antibody was added to the supernatant and shaken slowly overnight at 4 °C.
(7) The column was then washed 3 times with PBS.
(8) The column was added to the experimental group and the IgG group and shaken slowly on a horizontal shaker at 4 °C for 3–5 h.
(9) Following shaking, it was centrifuged at 2500 rpm for 3 min and the supernatant was discarded.
(10) The precipitate was then washed with protein lysis solution for 5 times.
(11) We added 20–40 μL loading buffer and boiled it together with the input group for 10 min.
(12) The sample was loaded on an SDS-PAGE at a concentration of 10%, and proteins were separated and transferred to PVDF membrane via blotting.

Anti-SPARCL1 antibody and protein A + G beads (Beyotime, Biotechnology) were used for SPARCL1 immunoprecipitation. IgG was used as a negative control. Cell lysates were resolved on a 10% SDS-polyacrylamide gel, and the gel was stained with Coomassie blue. The specific bands were cut out and sequenced using QE mass spectrometry to identify the interaction with SPARCL1. ITGB1 protein function was then positively and reversely verified using SPARCL1 and ITGB1 primary antibodies for Co-IP experiments.

### 2.7. Immunofluorescence

Culture medium was removed from the six-well plates. Next, cells were fixed with 4% paraformaldehyde for 10 min at 25 °C temperature, washed 3 times with PBST on a shaker, and blocked with 5% BSA in PBST for 1 h at 5 °C. After removal of the blocking solution, cells were incubated with primary anti-desmin antibody (dilution 1:50, Santa Cruz) diluted with 5% BSA in PBST (0.1% Triton X-100 in PBS) overnight at 4 °C. Then, cells were incubated with a FITC-conjugated secondary antibody diluted in 5% BSA in PBST for 1 h at 37 °C. Next, the cells were washed with PBST on a shaker, stained with the nuclear dye 4, 6-diamino-2-phenylindole (DAPI) for 5 min at 25 °C temperature, washed with PBST, and finally capped with an anti-fluorescence quencher (Beyotime Biotechnology, Shanghai, China). Fluorescence was captured using an inverted fluorescence microscope (BX43, OLYMPUS, Tokyo, Japan).

### 2.8. Antibodies and Chemicals

Rabbit anti-desmin (sc-14026) (at dilution 1:500; Santa Cruz Biotechnology, USA). Rabbit anti-SPARCL1 antibody (bs-6110R), rabbit anti-integrin beta 1 antibody (bs-0486R), rabbit anti-FAK antibody (bs-20735R), rabbit anti-paxillin antibody (bs-3539R), rabbit anti-phospho-FAK (Tyr397) antibody (bs-3159R), rabbit anti-phospho-paxillin (Tyr118) antibody (bs-3352R), rabbit anti-vinculin antibody (bs-6640R), rabbit anti-ARP2/3 subunit 1B antibody (bs-10563R), rabbit anti-CDC42 antibody (bs-3555R), rabbit anti-MyoD antibody (bs-2442R), rabbit anti-GAPDH antibody (bs-0755R) (all at dilution 1:500; Bioss Antibodies, China). These antibodies mentioned above were polyclonal antibodies, thus, they are compatible for protein detection in bovine.

### 2.9. Statistical Analysis

All data were obtained from at least three independent experiments. Results are expressed as means ± standard error of the mean. The myotube fusion rate was determined by dividing the number of nuclei in the fused myotubes by the total number of nuclei. The resulting myotube fusion rate was calculated by averaging the findings from five images using the ImageJ software. Differences between each two sets of data were analyzed using *t*-test. The greyscale scanning of protein bands in Western blotting was performed with the ImageJ software, and data were analyzed using GraphPad Prism. Immunofluorescence images were assessed with the ImageJ software. *P* values lower than 0.05 were considered statistically significant.

## 3. Results

### 3.1. SPARCL1 Interacts with ITGB1

**Figure 1:**
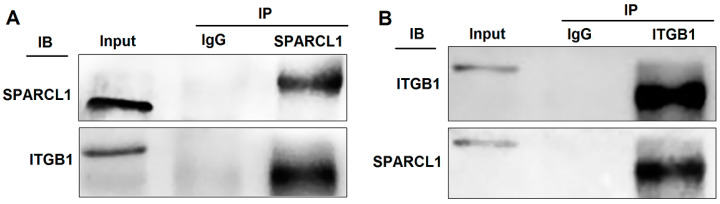
Secreted protein acidic and rich in cysteine like 1 (SPARCL1) interacts with integrin β1 (ITGB1). (**A**) Co-immunoprecipitation with an anti-SPARCL1 antibody followed by Western blotting with an anti-ITGB1 antibody. (**B**) Co-immunoprecipitation with an anti-ITGB1 antibody followed by Western blotting with an anti-SPARCL1 antibody. Input, IgG, and IP represent the positive control, negative control, and target experimental group, respectively.

Protein samples containing SPARCL1 were collected using co-immunoprecipitation. After gelation, the specific bands were sequenced using QE mass spectrometry. Interestingly, SPARCL1 could bind several integrin proteins, such as integrin alpha-3, ITGB1, and integrin alpha-V (Supplementary Table S1. The QE mass spectrometry results of the proteins bound with SPARCL1). Considering our previous results, ITGB1 was abundantly expressed and significantly increased during bovine MDSC differentiation. Therefore, we hypothesized that SPARCL1 may interact with ITGB1 and play a role in MDSC differentiation. Then, the anti-SPARCL1 antibody was used for immunoprecipitation followed by Western blotting with the anti-ITGB1 antibody (Figure 1A) or vice versa (Figure 1B). The co-immunoprecipitation results showed that SPARCL1 and ITGB1 interact with each other.

### 3.2. ITGB1 Influences MDSC Migration and Differentiation

**Figure 2:**
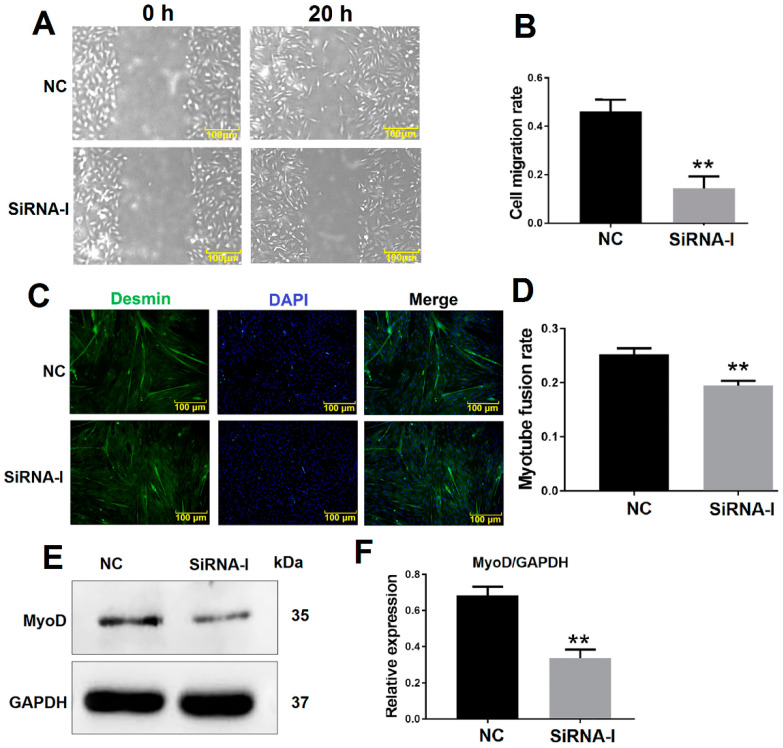
ITGB1 influences muscle-derived satellite cell (MDSC) migration and differentiation. (**A**) Cell scratch assay images 20 h after ITGB1 inhibition. (**B**) Quantification of the cell migration rate 20 h after ITGB1 inhibition as presented in (A). (**C**) Desmin immunofluorescence staining in MDSCs 48 h after ITGB1 inhibition. (**D**) Quantification of the myotube rate based on (C). (**E**) Western blotting results of MyoD expression 48 h after ITGB1 inhibition. (**F**) Quantification of the MyoD Western blotting results presented in (E). Scale bar = 100 μm. ** *p* < 0.01.

The bovine MDSC migration rate was decreased 20 h after ITGB1 inhibition as determined by a cell scratch assay (Figure 2A,B). Furthermore, desmin was detected by immunofluorescence to analyze the myotube fusion rate. ITGB1 inhibition reduced differentiation and was associated with decreased myotube fusion rate after 48 h ITGB1 siRNA treatment (Figure 2C,D). In addition, the protein levels of the early differentiation marker MyoD decreased 48 h after ITGB1 inhibition (Figure 2E,F). These results showed that ITGB1 may influence bovine MDSC migration and early-stage differentiation.

### 3.3. ITGB1 Inhibition Affects Downstream Protein Expression

**Figure 3:**
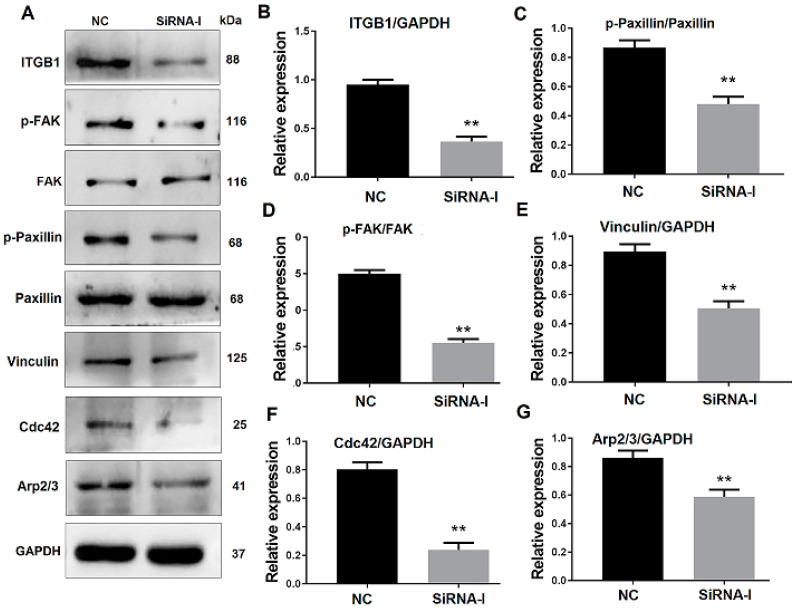
ITGB1 inhibition affects downstream gene expression. (**A**) Western blotting images of proteins related to the ITGB1-mediated signaling pathway at 48 h after ITGB1 inhibition. (**B**–**G**) Quantification of the ITGB1, p-FAK, p-paxillin, vinculin, Cdc42, and Arp2/3 Western blotting results presented in A. ** *p* < 0.01.

To verify the effect of ITGB1 on bovine MDSC migration, ITGB1 was inhibited by its siRNA. MDSCs were transfected with ITGB1 siRNA for 24 h, their differentiation was induced by 2% horse serum, and cells were collected 48 h after differentiation. Western blotting results showed that ITGB1 is decreased significantly after treatment with its siRNA fragment (Figure 3A,B). In addition, p-FAK, p-paxillin, vinculin, Cdc42, and Arp2/3 were all reduced, when ITGB1 was inhibited (Figure 3A,C–G). These results indicated that ITGB1 inhibition down-regulates downstream protein expression.

### 3.4. SPARCL1 Influences MDSC Migration and Differentiation

**Figure 4:**
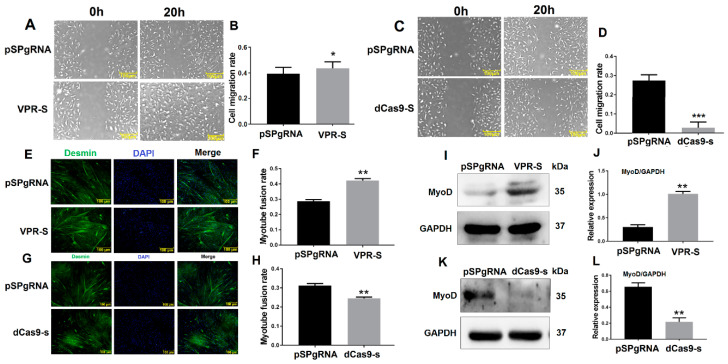
SPARCL1 influences MDSC migration and differentiation. (**A**,**C**) Cell scratch assay images at 20 h after SPARCL1 activation or inhibition. (**B**,**D**) Quantification of the cell migration rate at 20 h after SPARCL1 activation or inhibition according to (**A**,**C**). (**E**,**G**) Desmin immunofluorescence staining in MDSCs after SPARCL1 activation or inhibition. (**F**,**H**) Quantification of the myotube rate based on (**E**,**G**). (**I**,**K**) Western blotting images of MyoD expression after SPARCL1 activation or inhibition. (**J**,**L**) Quantification of MyoD from Western blotting results presented in (**I**,**K**). Scale bar = 100 μm. * *p* < 0.05, ** *p* < 0.01, *** *p* < 0.001.

SPARCL1 interacts with ITGB1, whereas ITGB1 affects MDSC migration; therefore, we speculated that SPARCL1 influences MDSC migration and early-stage differentiation. To test this hypothesis, SPARCL1 was inhibited or activated by a CRISPR system. The results of the scratch assay showed that MDSC migration rate is increased, when SPARCL1 is activated by its VPR vector (Figure 4A,B). As expected, the cell migration rate was decreased significantly at 20 h after SPARCL1 inhibition (Figure 4C,D). In addition, desmin staining confirmed that the myotube fusion rate is increased or decreased by SPARCL1 activation or inhibition (Figure 4E–H), respectively. Furthermore, MyoD was up-regulated or down-regulated significantly when SPARCL1 was activated or inhibited (Figure 4I–L) respectively. These results showed that SPARCL1 affects MDSC migration and early-stage differentiation.

### 3.5. SPARCL1 Affects an ITGB1-Mediated Signaling Pathway

**Figure 5:**
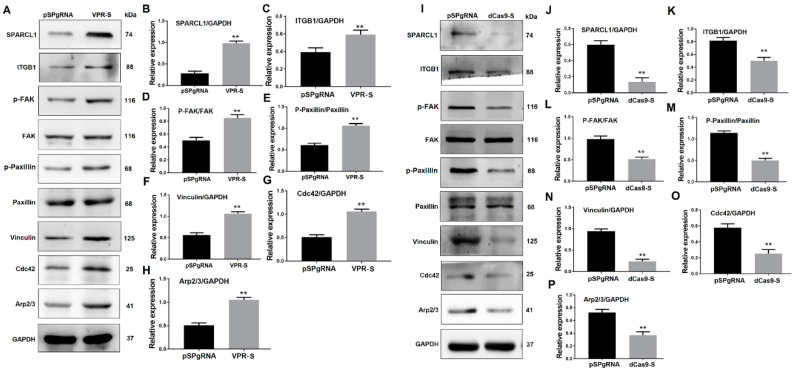
SPARCL1 affects an ITGB1-mediated signaling pathway. (**A**,**I**) Western blotting results for ITGB1, p-FAK, FAK, p-paxillin, paxillin, vinculin, Cdc42, and Arp2/3 after SPARCL1 activation or inhibition, respectively. (**B**–**H**) Quantification of the Western blotting results presented in (**A**). (**J**–**P**) Quantification of the Western blotting results presented in (**I**). ** *p* < 0.01.

To determine whether SPARCL1 affects MDSC migration and differentiation through ITGB1, SPARCL1 was activated or inhibited by a CRISPR system. Then, the expression of ITGB1 and its downstream genes was detected. Western blotting results showed that SPARCL1 activation up-regulates ITGB1 expression. In addition, p-FAK, p-paxillin, and vinculin were also significantly up-regulated 48 h after SPARCL1 activation (Figure 5A,H). Furthermore, the ITGB1 downstream genes Cdc42 and Arp2/3 were both increased at 48 h after ITGB1 activation. As expected, ITGB1, p-FAK, p-paxillin, vinculin, Cdc42, and Arp2/3 were all down-regulated after SPARCL1 inhibition (Figure 5I,P). Together, these results indicated that SPARCL1 influences an ITGB1-mediated cell signaling pathway.

### 3.6. SPARCL1 Influences Cell Migration and Differentiation through an ITGB1-Mediated Cell Signaling Pathway

#### 3.6.1. SPARCL1 Influences Cell Migration and Differentiation through ITGB1

**Figure 6:**
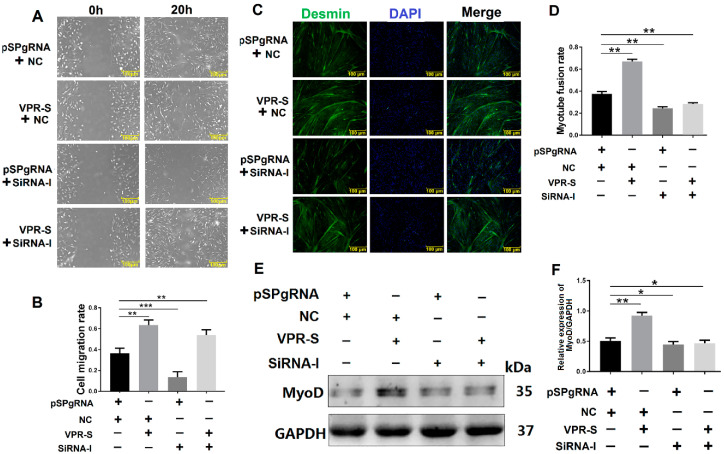
SPARCL1 influences cell migration and differentiation through ITGB1. (**A**) Cell scratch assay images after simultaneous SPARCL1 activation and ITGB1 inhibition. (**B**) Quantification of the cell migration rate based on (**A**). (**C**) Desmin immunofluorescence staining of MDSCs after SPARCL1 activation and ITGB1 inhibition. (**D**) Quantification of the myotube rate represented in (**C**). (**E**) Western blotting results of MyoD expression after SPARCL1 activation and ITGB1 inhibition. (**F**) Quantification of the MyoD Western blotting results presented in (**E**). pSPgRNA is the blank control for the SPARCL1 activation group, NC is the negative control for the ITGB1 siRNA interference group, VPR-S represents the SPARCL1 activation group, and siRNA-I represents the ITGB1 siRNA interference group. * *p* < 0.05, ** *p* < 0.01, *** *p* < 0.001; scale bar = 100 μm.

To explore whether SPARCL1 influences MDSC migration and differentiation through ITGB1, SPARCL1 was activated by a CRISPR system, whereas ITGB1 was simultaneously inhibited by siRNA. Then, the cell scratch assay was performed at 20 h after SPARCL1 activation and ITGB1 inhibition. The results showed that the cell migration rate is lower with SPARCL1 activation and ITGB1 inhibition than with SPARCL1 activation alone. This indicated that when ITGB1 is inhibited, cell migration is down-regulated, even though SPARCL1 is activated (Figure 6A,B). In addition, desmin staining confirmed that the myotube fusion rate and MyoD protein expression are both decreased by SPARCL1 activation and ITGB1 inhibition relative to that by SPARCL1 activation alone (Figure 6C–F). It implied that the promoting effect of SPARCL1 on MDSC early differentiation is attenuated by ITGB1 inhibition. Taken together, these results demonstrated SPARCL1 influence on bovine MDSC migration and early-stage differentiation through ITGB1.

#### 3.6.2. SPARCL1 Regulates an ITGB1-Mediated Signaling Pathway through ITGB1

**Figure 7:**
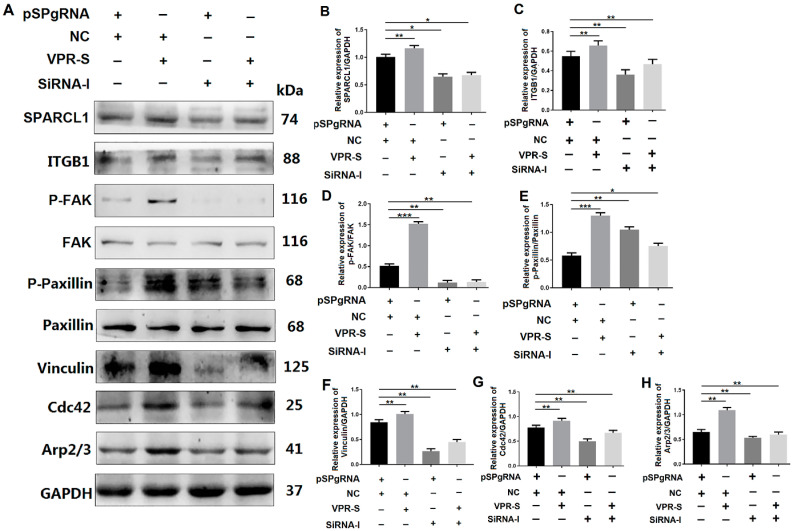
SPARCL1 regulates an ITGB1-mediated signaling pathway through ITGB1. (**A**) Western blotting results for ITGB1, p-FAK, FAK, p-paxillin, paxillin, vinculin, Cdc42, and Arp2/3 expression after SPARCL1 activation and ITGB1 inhibition. (**B**–**H**) Quantification of the Western blotting results presented in (**A**). pSPgRNA is the blank control for the SPARCL1 activation group, NC is the negative control for the ITGB1 siRNA interference group, VPR-S represents the SPARCL1 activation group, and siRNA-I represents the ITGB1 siRNA interference group. * *p* < 0.05, ** *p* < 0.01, *** *p* < 0.001.

To explore the mechanism, through which SPARCL1 affects MDSC migration and differentiation, Western blotting analysis was performed to ascertain whether SPARCL1 regulates the ITGB1-mediated signaling pathway through ITGB1. The expression of p-FAK, p-paxillin, vinculin, Cdc42, and Arp2/3 was decreased in the SPARCL1 activation and ITGB1 inhibition groups relative to that in the SPARCL1 activation alone group (Figure 7). These findings indicated that SPARCL1 cannot stimulate ITGB1 downstream protein expression owing to the inhibition of ITGB1. Therefore, SPARCL1 may regulate its downstream signaling pathways through ITGB1 and influence bovine MDSC migration and differentiation.

## 4. Discussion

SPARCL1 and SPARC and are both belong to SPARC family of acidic secretory glycoprotein. SPARC was reported to promote the differentiation of mouse myoblasts, such as the C2C12 and MM14 cell lines [22,23]. A recent report showed the genetic variant of SPARC gene was associated with growth traits in Chinese cattle. The SPARC gene was highly expressed in skeletal muscle tissue and a SNP site of loci in SPARC gene is significantly associated with cattle body [24]. It implied that SPARC may play important roles in bovine muscle development.

Protein BLAST result showed the percent identity of protein sequence between SPARCL1 and SPARC in bovine was approximately 61% based on the website of NCBI. Even though there are few reports about SPRCL1 regulated muscle development, we speculate that the mechanism of SPARCL1 regulates skeletal muscle differentiation might relate to its similarity of protein structure compared with SPARC. Then SPARCL1 was proved to regulate bovine MDSC differentiation in our previous study [8].

QE mass spectrometry was performed in this study to uncover the mechanism of SPARCL1 regulates bovine MDSC differentiation. According to the protein sequencing results, SPARCL1 may interact with several integrin proteins showed in Supplementary Table S1. SPARCL1, as one of a component of ECM, it assumed to combine with ITGB1 which located the cell membrane of bovine MDSC. Then the Co-IP assay was used to ascertain that SPARCL1 interacts with ITGB1 (Figure 1). ITGB1 is known in regulating cancer cell migration [25,26]. However, few reports related to the roles of ITGB1 in bovine MDSC differentiation. A recent study by our team showed that ITGB1 regulated bovine MDSC migration and differentiation mediated by platelet endothelial aggregation receptor-1 (PEAR1) [15]. We obtained the similar results in this study that ITGB1 regulated bovine MDSC migration by up-regulated several focal adhesion formation related proteins included p-FAK, p-paxillin, and vinculin (Figure 3). Moreover, we found SPRACL1 regulated the expression of p-FAK, p-paxillin, and vinculin through ITGB1 (Figure 5 and Figure 7). This suggested that SPARCL1 might play a role in focal adhesion assembly in bovine MDSC migration. 

Furthermore, Cdc42 activates the Arp2/3 complex to promote the formation of lamellipodia [27]; this in turn stimulates the leading edge cell membrane [16] to provide a microfilament-maintained driving force for the establishment of an adhesion between integrin and the ECM [17] and for the fixation of the cells in a new location. In this study, Arp2/3 and Cdc42 were also proved to be down-stream molecules influenced by SPARCL1 through ITGB1 (Figure 5 and Figure 7). Meanwhile, the cell scratch assay in this study demonstrated that SPARCL1 regulated bovine MDSC migration through ITGB1 (Figure 2, Figure 4, Figure 6). However, the more details of SPARCL1 interact with ITGB1 to regulate bovine MDSC migration remains deeply exploration.

In this study, we also determined the protein expression of the differentiation marker MyoD, which is a major regulator of skeletal muscle progenitor cells specialized in myoblasts. Satellite cells lacking MyoD and Myf5 (double knockout) remain aging in uninjured muscles; however, damaged muscles cannot regenerate [28]. MyoD is not only related to the specialization of myoblasts but is also a very important early differentiation factor, which can up-regulate p21 and Rb and withdraw cells from the cell cycle into terminal differentiation [29]. In this study, MyoD expression was also affected by the inhibition of ITGB1 (Figure 2). Even though integrin/FAK pathway is required for myoblast differentiation by regulating the expression of MyoD and Cdc42 [14], we have no direct evidence that ITGB1 regulates MyoD expression in bovine MDSC differentiation. 

In addition, it should be emphasized that desmin is an intermediate filament protein, it specifically expressed in myocytes [30]. Desmin is generally stained for the measurement of myotube fusion during cell fusion. In this study, bovine MDSCs were induced to differentiation at 48 h. MDSCs were analyzed at an early-stage of differentiation. There was not enough time for desmin expression; therefore, the thick myotubes are not shown in this study. 

Overall, SPARCL1 affects bovine MDSC migration by influencing the formation of focal adhesions and the expression of proteins involved in migration. Migration is an important step for myoblast induction to differentiation. This can explain why SPARCL1 and ITGB1 effects on bovine MDSC migration and early differentiation occur simultaneously.

**Figure 8:**
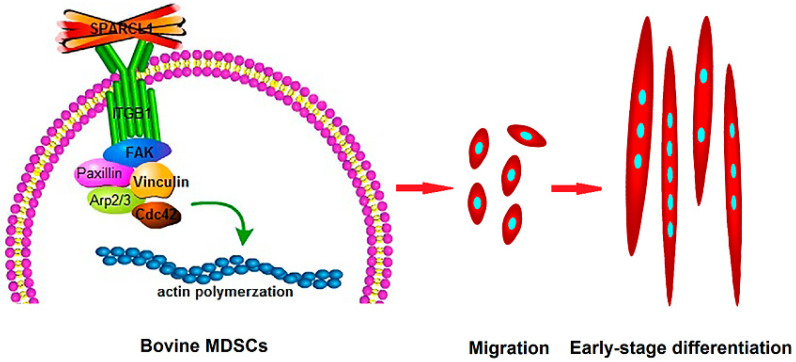
Proposed model for SPARCL1 regulation of bovine MDSC migration and differentiation through ITGB1. As an extracellular matrix protein, SPARCL1 interacts with the transmembrane receptor ITGB1 and regulates changes in the expression of adhesion-plaque-associated proteins, such as ITGB1, p-FAK, FAK, p-paxillin, paxillin, and vinculin to affect the formation of focal adhesions. The Cdc42 and Arp2/3 complex was also affected by SPARCL1 through ITGB1. Further, bovine MDSC migration was stimulated by SPARCL1, and cells gathered and apparently expressed MyoD to begin an early-stage differentiation.

In conclusion, SPARCL1 interacts with ITGB1 and influences cell migration and early differentiation through an ITGB1-mediated signaling pathway (Figure 8). These findings provide a new ideological and theoretical basis for the identification of the mechanism through which SPARCL1 regulates bovine MDSC differentiation. Furthermore, they provide new insights for future investigation of the mechanism through which ECM regulates bovine MDSC migration and differentiation. This may also be helpful for identifying strategies to improve the muscle quality of cattle.

## 5. Conclusions

SPARCL1 influences bovine MDSC migration and early-stage differentiation through ITGB1-mediated signaling pathway. This study will be helpful to reveal the mechanism of SPARCL1 regulates skeletal muscle differentiation and development.

## Supplementary Materials

The following are available online at https://www.mdpi.com/2076-2615/10/8/1361/s1, Table S1: The QE mass spectrometry results of the proteins bound with SPARCL1.
